# GCTRNet: global context-gated temporal refinement for short-window ECG-based premature ventricular contraction detection

**DOI:** 10.3389/fphys.2026.1979923

**Published:** 2026-10-14

**Authors:** Manhong Shi, Yuqing Yang, Jianguo Chen

**Affiliations:** College of Information and Network Engineering, Key Laboratory of Intelligent Information Processing, Anhui Science and Technology University, Bengbu, China

**Keywords:** BLSTM, electrocardiogram, global contextual refinement, inter-patient classification, premature ventricular contraction

## Abstract

**Background:**

Premature ventricular contractions (PVCs) are one of the most prevalent ventricular arrhythmias and therefore require a method for reliably identifying PVCs from short ECG strips, through an automated system capable of identifying PVCs and evaluating the burden of PVCs. While technology continues to advance, automatic identification of PVCs from very short ECG recordings remains problematic due to significant variability in PVC morphology across various patients as a result of the limited amount of information available from ECG data.

**Methods:**

To overcome this difficulty, the Global Context-Gated Temporal Refinement (GCTR) module has been created which enhances bidirectional long short-term memory (BLSTM) representations by incorporating segment-level contextual information using context gating and residual learning. The GCTR module was integrated into a convolutional network architecture termed GCTRNet. GCTRNet was evaluated on the MIT-BIH Arrhythmia Database using a patient-independent partition. The frozen model was further evaluated on a predefined subset of CPSC2021 comprising 210 recordings from 105 patients.

**Results:**

The frozen seed-2024 model of GCTRNet, when tested on MITDB data, reached an accuracy of 99.65%, a sensitivity of 96.82% and a specificity of 99.91%. The same frozen model achieved performance on the CPSC2021 subset with 97.83% accuracy, 84.75% sensitivity, and 98.11% specificity and an AUPRC of 80.49%. In addition, GCTRNet outperformed matched comparison models based on BLSTM architecture in many metrics of evaluation, thus supporting the potential of the temporal refinement method proposed.

**Conclusions:**

GCTRNet provides an effective temporal refinement approach for patient-independent short-window PVC detection and shows potential for cross-database application.

## 1 Introduction

Premature ventricular contractions (PVCs) are one of the most common arrhythmias present not only in healthy people but also in people suffering from heart diseases. Although occasional PVCs have been regarded as clinically insignificant, the occurrence of PVCs frequently or continuously may lead to decreased function of the left ventricle, development of PVC-induced cardiomyopathy, heart failure, and increased chance of adverse cardiovascular events among vulnerable groups of patients (Huizar et al., 2019; Marcus, 2020; Zeppenfeld et al., 2022). The estimated prevalence of PVCs varies significantly based on the method of monitoring and length of observation: standard resting ECG allows to identify PVCs in around 1-4% of the population during 10–15 seconds of monitoring, while longer ambulatory methods such as 24–48 hour monitoring can detect PVCs at the rates between 40% and 75% (Ahn, 2013; Zeppenfeld et al., 2022). The advance of technology has made portable ECG devices and systems for long-term monitoring more accessible since they generate large volumes of data for heartbeats, which are being used in practice nowadays for screening of heart diseases. Therefore, the automation of PVC detection becomes crucial for the analysis of ECG and long-term heart observation both in clinical settings and for the purposes of clinical decision support using computer technology (Yu et al., 2021).

The older paradigm of detecting PVCs was heavily dependent on the application of rule-based systems and features derived from manual processing. Rule-based methods could be perceived as methods for formulating diagnostic requirements for PVC detection based on RR intervals, QRS duration, morphology of waveforms, heart rate, and other significant information presented by ECG (Wieben et al., 1999; Xie et al., 2019). However, the emergence of machine learning has given researchers the opportunity to combine engineered features with statistical classifiers to enhance recognition efficacy. Some commonly used descriptors are represented by the following: temporal intervals, morphologic measures, wavelet coefficients, principal component representations, dynamic time warping features, entropy and nonlinear dynamic features, while the most notable classifiers include support vector machine, random forest, K-nearest neighbor (KNN) classifier, decision tree, and shallow neural networks (Mar et al., 2011; Saenz-Cogollo and Agelli, 2020; Yu et al., 2021; De Marco et al., 2022; Mazidi et al., 2022; Sarshar and Mirzaei, 2022). For instance, Mazidi et al. coupled tunable Q-factor wavelet decomposition with statistical feature extraction and standard classifiers in order to recognize PVCs (Mazidi et al., 2022). Mar et al. combined ECG-feature selection with both temporal and morphologic descriptors (Mar et al., 2011), while Saenz-Cogollo and Agelli evaluated informative handcrafted features using an inter-patient random forest approach (Saenz-Cogollo and Agelli, 2020). Yu et al. adopted a deep metric learning approach combined with KNN for PVC detection (Yu et al., 2021). Overall, the aforementioned methodologies facilitated satisfactory results, but their performance is still very much reliant on the efficiency of signal preprocessing, fiducial-point localization, feature engineering, and the involvement of domain experts. Additionally, the separation of feature extraction from classification prevents the entire detection process from being optimized within a unified framework, which may limit the overall performance of these approaches (Saenz-Cogollo and Agelli, 2020; Yu et al., 2021; De Marco et al., 2022; Sarshar and Mirzaei, 2022).

Deep learning has shifted ECG automated analysis from the utilization of hand-crafted features to the networks’ capability to learn representations directly from signals in the form of raw or minimally processed data (Ansari et al., 2023). CNNs excel in the extraction of local waveforms; on the other hand, recurrent architectures, for instance, LSTM and Bi-LSTM networks, deal well with the temporal dependencies of ECGs (Acharya et al., 2017; Chen et al., 2020). The fact that these network types complement each other led to the frequent use of CNN-LSTM and CNN-BLSTM architectures for beat and arrhythmia classification. Moreover, some recent studies adopted attention mechanisms, Transformer-based encoders, and multi-scale feature extraction to further improve the representation of ECG signals and acquire ECG features across different receptive fields (Vaswani et al., 2017; Kachuee et al., 2018; Shin et al., 2023). In relation to the detection of PVCs, recent research has started giving preference to more realistic evaluation approaches instead of relying solely on beat-wise assessment. To perform an end-to-end analysis of Holter ECG, Kraft et al. developed an end-to-end model and tested it on several databases, showing that large-scale PVC detection is feasible (Kraft et al., 2023). Wang et al. created PVCNet, which applied sequence-level heartbeat labels and loss redesign under the inter-patient evaluation protocol and later was tested on an independent external database (Wang et al., 2024). Ma et al.’s study presented DPFNet, which combined one-dimensional heartbeat representations with two-dimensional scalograms to enhance the quality of PVC identification (Ma et al., 2026). Overall, these studies showed the recent progress in automatic PVC detection and also demonstrated the need to test the models on different patients and ECG databases.

Nevertheless, recognizing PVCs from short ECG windows remains a challenging task in feature representation. While multi-scale convolutional encoders can extract complementary local morphological features, and BLSTM networks can encode bidirectional temporal dependencies, the hidden states output by BLSTM are commonly directly pooled. In this setup, the segment-level summary is generated only after temporal encoding, and is not fed back into the hidden-state sequence. Thus, a simple mechanism that integrates segment-level context with individual recurrent states before the pooling step may help improve the representation of short ECG segments for classification tasks.

In addition, PVC-related characteristics, such as QRS widening, altered amplitude, slope, and post-QRS responses, may vary considerably across patients. Besides developing new methodologies, having a solid experimental design is another key factor in ECG classification. When random splitting occurs on the beat basis, it leads to the same recording appearing in both the training and testing datasets. This can cause some amount of information leakage resulting in over-estimation of performance. In addition to that, selecting thresholds on the test set can lead to optimistically biased performance estimates. In turn, cross-database evaluation adds variability due to different orientations of leads, polarities of waveforms, noise levels, signal scaling, sampling frequencies, acquisition systems, and types of patients. To implement a valid evaluation, it is necessary to ensure independent data partitioning from patients prior to the segmentation process, validation-based choice of model checkpoints and threshold settings, the ability to evaluate new data using an already-fixed model, and a clear separation between confirmatory and exploratory external tests.

In response to this challenge, the current study proposes a multi-scale BLSTM architecture named GCTRNet, which contains a post-encoding Global Context-Gated Temporal Refinement (GCTR) module. The multi-scale encoder extracts complementary local morphological features before BLSTM bidirectional encoding. GCTR operates after BLSTM encoding by summarizing the hidden-state sequence into a segment-level context vector and then projecting and broadcasting this vector along the temporal dimension. The broadcast context is combined with each hidden state through an adaptive gate and a residual update with learnable scaling. The refined hidden-state sequence is then pooled for classification. In conclusion, there are four key takeaways from this study:

1. The Global Context-Gated Temporal Refinement (GCTR) has been designed to refine the hidden states of BLSTM with the help of segment-wise contextual feedback, adaptive contextual gating, and trainable residual scaling.
2. GCTR was incorporated into a multi-scale BLSTM architecture to produce GCTRNet and was compared with the matched MS-BLSTM, SE-like, and temporal-attention models in order to assess the impact of the contextual refinement procedure applied after BLSTM.
3. Two independent convolutional models, namely FCN1D and ResNet1D, were also added to the comparison. All six models were evaluated using the same patient-fixed MITDB partition with different random seeds. The role of GCTR components and BLSTM hidden-state dimension was investigated using ablation and sensitivity analyses.
4. The experiments were run using the subject-independent MITDB evaluation setting, followed by frozen-model external validation on a predefined subset of CPSC2021 comprising 210 recordings from 105 patients.

## 2 Materials and methods

### 2.1 Dataset, partitioning, and preprocessing

Using short-window ECG segments, we classified the segments as premature ventricular contractions (PVCs) or normal beats using a binary classification model. We used the MIT-BIH Arrhythmia Database (MITDB) in developing and evaluating the model (Goldberger et al., 2000; Moody and Mark, 2001). The dataset comprises 48 Holter ECG recordings of approximately 30 minutes’ duration. These were collected from 47 individuals and were sampled at 360 Hz in two channels. Four records with paced beats (102, 104, 107, and 217) were excluded as in previous studies of PVC classification, resulting in 44 ECG recordings being analyzed from 43 individuals. The MLII lead was selected by lead name; it corresponded to channel 1 for record 114 and channel 0 for the remaining 43 recordings.

For this study, the 44 records were then separated into three datasets for training, validation, and testing purposes using a patient-independent, record-wise partition. All ECG segments from the same recording were kept in the same dataset to avoid data leakage. Since recording 201 and recording 202 originated from the same patient/subject, they were both assigned to the training dataset, leaving no overlap in recordings or subjects among the training, validation, and test datasets. As a result, the training, validation, and test datasets were fixed partitions composed of 31 training, 6 validation, and 7 test records, respectively, as detailed in **Table 1A**.

**Table 1A:** Dataset composition under the patient-fixed MITDB partition.

Subset	Records	ECG segments	PVC	Normal (N)
Training	31	60,051	5,084	54,967
Validation	6	8,007	682	7,325
Test	7	13,300	1,133	12,167
Total	44	81,358	6,899	74,459

Records denote the original MITDB ECG recordings assigned before segment extraction. The training, validation, and test record sets were mutually disjoint. Records 201 and 202, which originated from the same subject, were both assigned to the training set.

**Table 1B:** Characteristics of MITDB and the selected CPSC2021 external subset.

Dataset	Study subset	ECG source and selected lead	Normal/PVC segments; PVC prevalence
MITDB	44 records, 43 subjects	Two-channel Holter; MLII; 360→250 Hz	74,459/6,899; 8.48% PVC
CPSC2021	210 selected records, 105 patients; 209 contributing records	12-lead Holter or 3-lead wearable ECG monitoring devices; released Lead II	392,699/8,313; 2.07% PVC

Age and sex were not reported in the publicly available CPSC2021 metadata and therefore could not be compared with MITDB.

To develop the MITDB study dataset, first the recordings were resampled from 360 Hz to 250 Hz, and the annotation positions were mapped to the resampled signals. All ECG signals were processed with a standard sampling rate of 250 Hz. So each 2-second portion consisted of 500 samples, thus allowing the preprocessing step and network input sizes for both datasets to be identical. Beat labels and beat positions were taken from the reference annotations supplied with MITDB. No manual relabeling of beats was performed in this study. A two-second (500-point) window was extracted around each N (normal) or V (PVC) annotation, with the annotated beat positioned at the center of the window at the 1.0-second mark. If the segments crossed the edges of recordings, they were eliminated. Since every window was centered on the annotated beat, the adjacent windows obtained from the same recording could overlap. However, all recordings were divided into training, validation, and testing sets prior to segment extraction and all portions made from the same recording remained in one specific set. This was why there was no overlapping of recordings among training, validation, and testing sets, respectively. All segments with undefined values and segments with a standard deviation that was lower than 1×10^−8^ were excluded from further analysis after the noise reduction process had been concluded. In this particular case, there was no other filtration of signal quality. Finally, the amount of segments that were obtained for analysis from the MITDB database was 81,358 in total, consisting of 74,459 of normal-beat segments and 6,899 PVC segments altogether. All extracted segments were denoised using the four-stage discrete wavelet transform based on the Daubechies-6 wavelet and were then normalized using z-score normalization and stored as tensors with a shape of [B, 1, 500], where B is the batch size of relevant sessions.

Independent external validation of these results was obtained using the China Physiological Signal Challenge 2021 (CPSC2021) public dataset (Wang et al., 2021). CPSC2021 was selected because it provides an independent patient cohort recorded using 12-lead Holter or 3-lead wearable ECG monitoring devices, while retaining publicly available Lead II waveforms and beat annotations, thereby enabling evaluation of the frozen model under cross-database domain shift. The public dataset consists of 1,436 recordings from 105 patients, acquired using either a 12-lead Holter or a 3-lead wearable ECG system; the publicly released data contain the waveforms for Lead I and Lead II at 200 Hz. Each recording was only eligible for inclusion when the requisite waveform, header, and annotation files were readable, the annotation file was not empty, and Lead II was specified in the header. Of the 1,436 CPSC2021 recordings, 1,425 met these eligibility requirements. For each patient, two eligible recordings were selected at random without replacement using the fixed random seed of 2024, producing 210 selected recordings. Of these, 209 had at least one eligible N or V segment available.

For each selected recording, Lead II was resampled from 200 to 250 Hz, and the annotation locations were mapped to the resampled signal. Only annotations labeled N or V were then retained for further data analysis. As with MITDB, the beat labels and locations were obtained directly from the original annotations supplied by the CPSC2021 database. No manual relabeling was performed. Next, a 500-point window of data was extracted around each retained annotation, using the same 2-second, beat-centered definition as that used for the MITDB recordings. At this point, all extracted windows underwent window-level wavelet denoising, finite-value and standard-deviation checks, and z-score normalization, as done for the MITDB dataset. No additional signal-quality thresholds were applied to the CPSC2021 segments. Neither age nor sex was available in the publicly released CPSC2021 metadata; therefore, these characteristics were not used in between-dataset comparisons. The overall study design and representative ECG preprocessing steps are illustrated in **Figure 1**.

**Figure 1:**
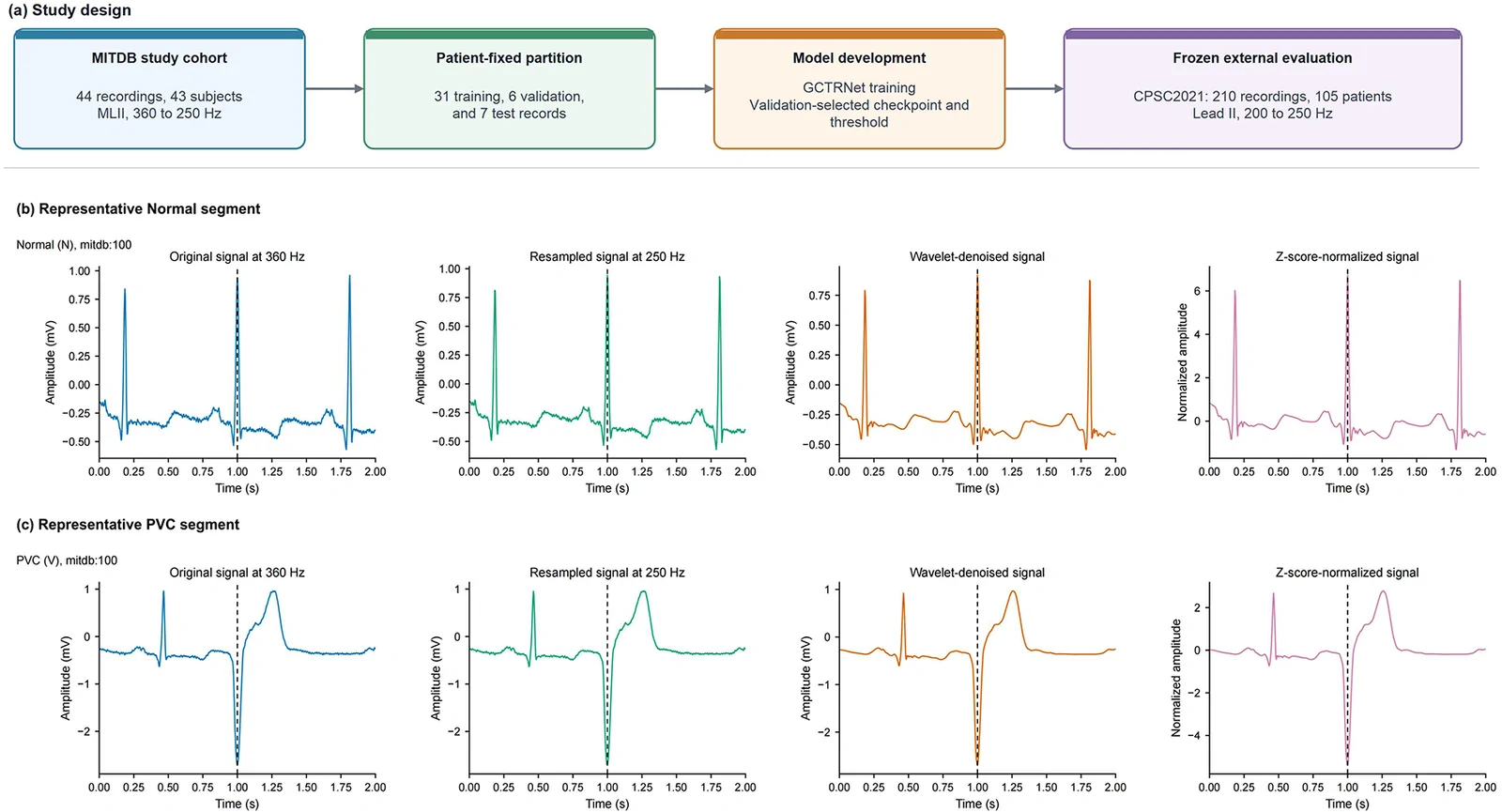
Study design and representative ECG preprocessing steps. **(a)** Patient-independent record-wise partitioning of MITDB and frozen-model external evaluation on CPSC2021. Representative **(b)** normal-beat and **(c)** PVC segments are shown at the original sampling frequency, after resampling to 250 Hz, after four-level db6 wavelet denoising and reconstruction, and after per-window z-score normalization.

### 2.2 GCTRNet architecture

The complete GCTRNet detector can be visualized in **Figure 2**; it includes several features such as multi-scale morphology encoding, BLSTM for temporal encoding, GCTR for refinement, temporal mean pooling, and binary classification.

**Figure 2:**
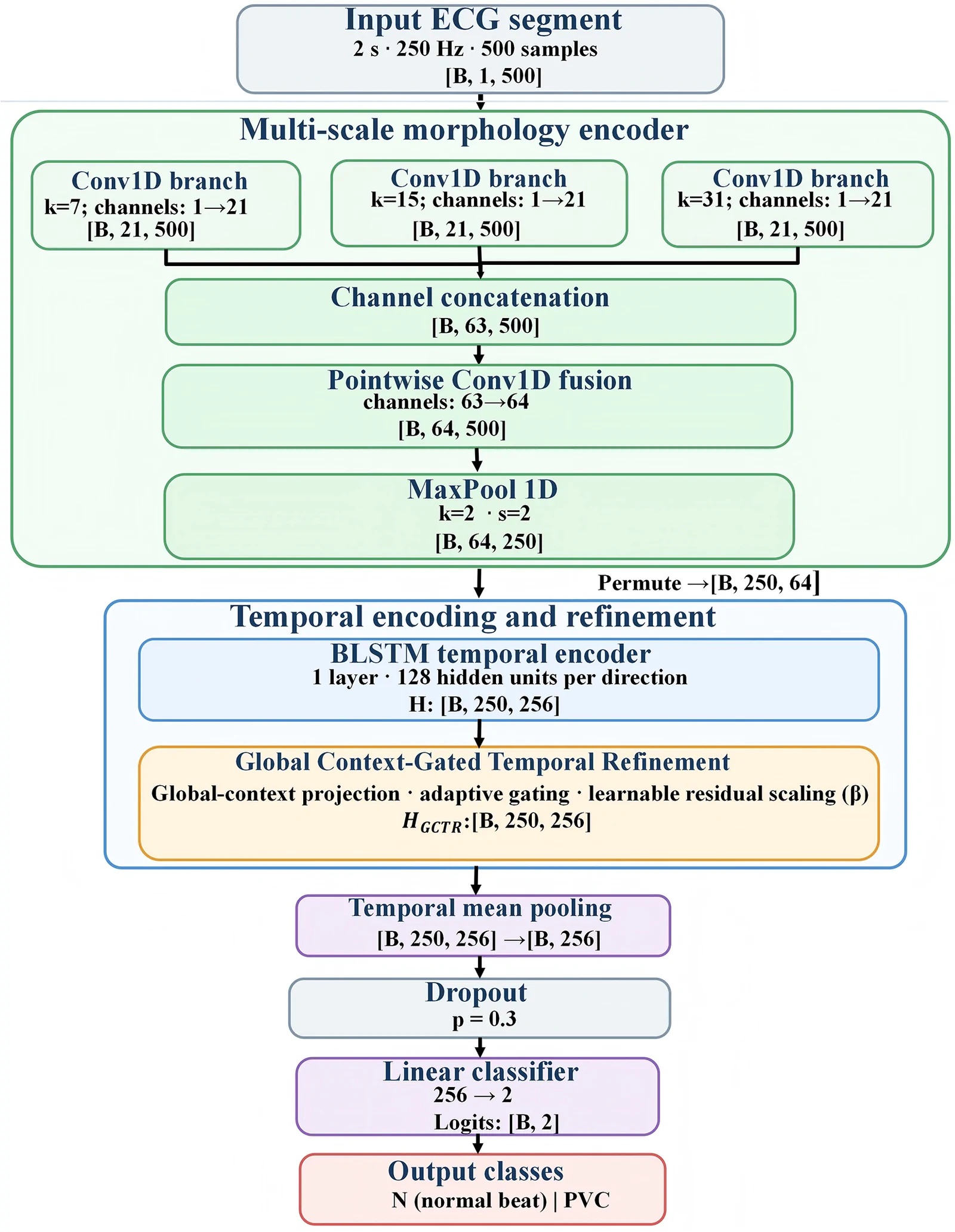
Overall architecture of GCTRNet for short-window PVC detection.

#### 2.2.1 Multi-scale morphology encoder

Given a 2-s single-lead ECG segment:


$$X\in R^{B\times 1\times 500},$$

Three parallel branches of one-dimensional convolutions with kernel sizes of 7, 15, and 31 samples are used to obtain receptive fields of about 28, 60, and 124 milliseconds at 250 Hz. These scales allow us to identify complementary slopes and sharp transitions (QRS-scale) and wider peri-beat waveform structure. Morphology is represented by the three convolutional branches, but they do not change the ordering of the subsequent BLSTM hidden states.

All three branches are stride-1 with same padding and map a single input channel (500 samples long) to 21 feature channels. The three branches are concatenated on a channel-wise basis resulting in 63 channels, subsequently fused by a 1 × 1 convolution to form 64 channels. Max pooling with kernel size 2 and stride 2 is then used to reduce the sequence length to 250, thus producing [B, 64, 250], which is permuted to [B, 250, 64] as input to the BLSTM.

The resulting feature sequence is:


$$\text{F}=f_{MS}\left(X\right),$$

with:


$$\text{F}\in R^{B\times 250\times 64}.$$

#### 2.2.2 BLSTM temporal encoding

Before temporal refinement, the extracted ECG features are processed by a bidirectional long short-term memory network (BLSTM) to capture forward and backward temporal dependencies (Hochreiter and Schmidhuber, 1997; Schuster and Paliwal, 1997).

Given the input feature sequence:


$$\text{F}\in R^{B\times T\times C}$$

the BLSTM produces the hidden-state sequence:


$$H=f_{\text{BLSTM}}\left(F\right),$$

where:


$$H\in R^{B\times T\times D}.$$

For the implementation of the model shown in **Figure 2**, the BLSTM has 128 hidden units in each direction (i.e., 128 in the forward direction and 128 in the backward direction), yielding a hidden-state sequence of 250 temporal positions with D = 256. The impact of D on performance was evaluated by setting D to 128, 256, and 512 while keeping all aspects of data partitioning, preprocessing, the random seed, and training fixed.

In **Figure 3**, the internal structure of the GCTR module is shown; this module is used to refine the BLSTM hidden-state sequence prior to temporal pooling. Three components comprise the GCTR: global-context construction, adaptive context gating, and learnable residual refinement. The GCTR constructs a segment-level context vector from the normalized BLSTM states. The segment-level context vector is projected and broadcast over time, and then introduced at each temporal location through adaptive gating and residual refinement before output normalization.

**Figure 3:**
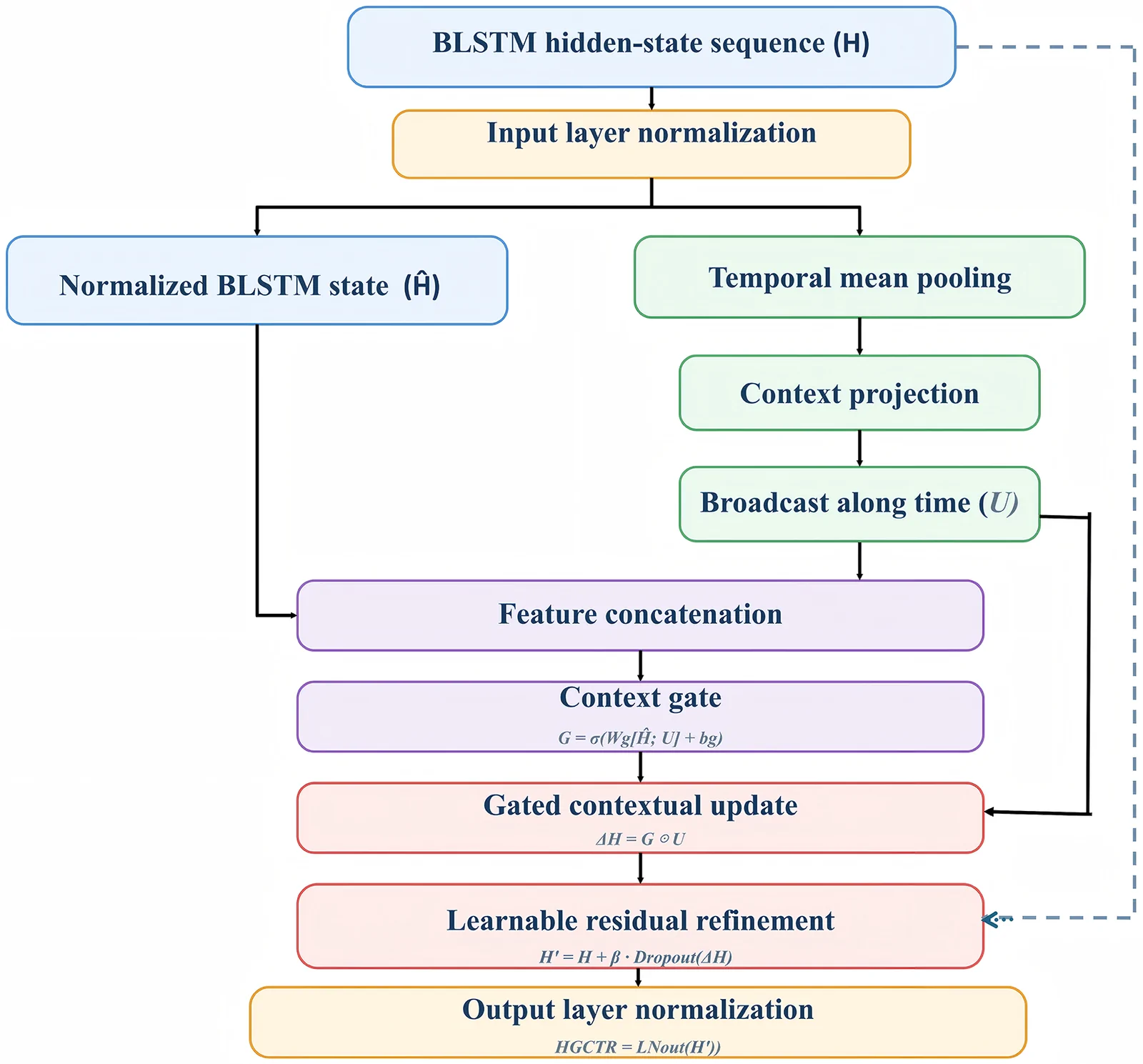
Global Context-Gated Temporal Refinement (GCTR) module.

#### 2.2.3 Global-context construction

When the BLSTM output H is available, layer normalization is performed first:


$$\hat{\text{H}}=\text{L}{\text{N}}_{\text{i}\text{n}}\left(\text{H}\right).$$

The context vector is formed by means of temporal aggregation:


$$\text{c}=\frac{1}{\text{T}}\sum_{t=1}^{T}{\hat{\text{H}}}_{\text{t}},$$

where


$$\text{c}\in R^{\text{B}\times \text{D}}$$

shows the overall characteristics of the segment.

Then, the context vector is subjected to a non-linear transformation:


$$\text{u}=\text{GELU}\left(\text{c}{\text{W}}_{\text{c}}+{\text{b}}_{\text{c}}\right),$$

where *W_c_* and *b_c_* are learnable parameters. The projected context vector *u* is then broadcast along the temporal dimension:


U=Broadcast(u),

resulting in:


$$\text{U}\in {\text{R}}^{\text{B}\times \text{T}\times \text{D}}.$$

GCTR differs from ordinary temporal pooling because the global context vector is fed back to every temporal position, allowing the hidden states to be refined using segment-level information before pooling.

#### 2.2.4 Context gating and adaptive residual refinement

Due to the differences in contextual relevance, GCTR introduces a context gate that ensures appropriate injection of context.

The normalized BLSTM representation is concatenated with the broadcast context:


$$\text{Q}=\left[\hat{\text{H}};\text{U}\right].$$

The equation for the context gate is:


$$\text{G}=\text{σ}\left(\text{Q}{\text{W}}_{\text{g}}+{\text{b}}_{\text{g}}\right),$$

where $G\in R^{B\times T\times D}$, and σ(·) is the sigmoidal function used in the gating operation.

The gate does not replace the BLSTM state directly; instead, it controls how much global-context information is introduced at each temporal position. The gated contextual update is then calculated as:


ΔH=G⊙U,

where ⊙ denotes element-wise multiplication.

A learnable residual refinement strategy is then applied:


$$H_{res}=H+\beta \cdot \mathrm{Dropout}\left(\Delta H\right),$$

where *β* is a learnable scalar which regulates the overall strength of contextual refinement. It was initialized to 0.01. This small initial value allowed GCTR to begin close to the original BLSTM representation while still permitting gradients to pass through the refinement branch.

Finally, output layer normalization was applied:


$$H_{GCTR}={\mathrm{LN}}_{\mathrm{out}}\left(H_{res}\right).$$

Using this approach, GCTR is able to preserve the original BLSTM representation while adaptively controlling the strength and position of contextual refinement.

#### 2.2.5 Temporal pooling and classification

Temporal mean pooling is then performed to obtain a segment-level representation:


$$\text{z}=\frac{1}{\text{T}}\sum_{t=1}^{T}{\text{H}}_{\text{GCTR},\text{t}}.$$

The final binary classification logits are then generated through dropout and a linear classifier:


$$\text{o}=\text{Dropout}\left(\text{z}\right){\text{W}}_{\text{o}}+{\text{b}}_{\text{o}}.\;$$

To enable controlled comparison, the MS-BLSTM baseline makes use of the same multi-scale convolutional encoder, BLSTM encoder, temporal pooling approach, dropout technique, and classifier, but does not utilize the GCTR process. Thus, the performance differences between MS-BLSTM and GCTRNet can be attributed to the GCTR operation.

### 2.3 Comparator configurations

The controlled comparison comprised four models based on the BLSTM architecture. The MS-BLSTM model consisted of the same multi-scale convolutional encoder as GCTRNet, BLSTM layer, temporal mean pooling layer, dropout layer, and classifier; however, it excluded the GCTR module. Two additional BLSTM models had either the temporal attention mechanism (Yang et al., 2016) or an SE-like channel-wise attention mechanism substituted for the GCTR module (Hu et al., 2018). This allowed the effects of the post-BLSTM refinement methods to be isolated while holding all other components of the network constant.

The controlled comparison also included two independent convolutional baselines (FCN1D and ResNet1D). The FCN1D baseline comprised three sequential convolutional layers followed by global average pooling and a linear classifier, while the ResNet1D baseline was a 1D version of a ResNet18-like architecture with four residual stages. Neither of the independent convolutional models included a recurrent layer, attention mechanism, SE block, GCTR module, transformer architecture, or manually designed feature extraction methods for ECGs. All six models in the controlled comparison were trained and evaluated using the same patient-fixed partition, the same preprocessed input segments, the same training procedure, and the same validation-based checkpoint and classification-threshold selection procedures.

### 2.4 Training, checkpoint selection, and thresholds

There was no application of K-fold cross-validation in this study. The comparison models that were specified earlier and the variants were evaluated utilizing the seeds 42, 2024, and 3407, while maintaining the patient-fixed data partition and the other conditions of the experiment unchanged. In other words, the values of these seeds were determined before comparison of the models, and they were not determined considering the results of their performance. All the models were trained using unweighted cross-entropy loss with AdamW (Loshchilov and Hutter, 2019), with a batch size of 64, a learning rate of 3 × 10^−4^, a weight decay parameter of 1 × 10^−4^, and a dropout rate established at 0.3. All models were trained using standard mini-batches for a maximum of 50 epochs, using early stopping and a learning-rate schedule based on the validation F1-score. No class weights, weighted sampling, over- or under-sampling methods, techniques for class-balanced batching, sample duplication, or data augmentation of any kind were used. The operating thresholds and checkpoints were selected only from the validation set and held fixed for evaluation of the final test set. The only seed for the hidden-dimension sensitivity analysis was seed 2024.

The ablation models were built on the same multi-scale encoder, BLSTM, temporal pooling, classifier, data partitions, and training processes; however, they differed as noted below. The LN-only ablation model excluded global-context creation, adaptive gates, and residual scaling, but used only the layer normalization of the output layer of the post-BLSTM model. The w/o Gate ablation model had global-context projection and learnable residual scaling, but did not apply adaptive gates. The model with no learnable scaling utilized both the context and gating methods; however, it set the scaling coefficient β = 1.

### 2.5 Evaluation metrics

Class imbalance complicated the treatment of normal beats (as the negative class) and PVCs (as the positive class), so we evaluated the performance of the models using multiple metrics—Accuracy (ACC), Sensitivity (SEN), Specificity (SPE), Precision, F1-Score, Area Under the Precision–Recall Curve (AUPRC), and Matthews Correlation Coefficient (MCC), accompanied by complete confusion matrices detailing true positives (TP), true negatives (TN), false positives (FP), and false negatives (FN).

SEN measures the proportion of PVCs that are correctly identified, while SPE measures the proportion of normal beats that are correctly identified. The precision is calculated using the formula TP/(TP + FP), and the F1-Score provides a balance between SEN and precision at the threshold that is chosen by maximizing the F1-Score on the validation set. To evaluate performance under class imbalance, AUPRC was calculated using the probabilities for PVC detection. Finally, the Matthews Correlation Coefficient incorporates all four components of the confusion matrix and gives a single score ranging from −1 to 1.

### 2.6 Explainability and representation analysis

Three complementary sets of analyses were performed to characterize the behavior of the model: (1) The compactness and separability of features at high dimensionality before and after GCTR were examined, where t-SNE was used solely for the purpose of visualization; (2) The mapping of TN, TP, FP, and FN outcomes and the representative classification error waveforms; (3) A temporal attribution at the input level with IG. Note that these three analyses are all descriptive and are not to be interpreted as demonstrating cause and effect through physiological explanations for how this occurs.

#### 2.6.1 Feature representation analysis

Pooled 256-dimensional representations were collected from the frozen seed-2024 GCTRNet prior to (Pre-GCTR) and immediately after (Post-GCTR) GCTR. The Pre-GCTR features were derived from the temporal average of the raw BLSTM hidden state sequence while the Post-GCTR features were derived from the temporal average of the refined sequence. The silhouette coefficient [higher = better (Rousseeuw, 1987)], Davies-Bouldin index [lower = better (Davies and Bouldin, 1979)], centroid distance, within-class dispersion, and the ratio of centroid distance to within-class dispersion were calculated for raw (unnormalized), L2-normalized, and z-score normalized features. For visualization purposes, the L2 normalized features for all 13,300 test samples were projected into two dimensions using t-SNE with a random seed of 42; however, the conclusions derived from this analysis were based solely on the original high-dimensional representations, not on the visual spacing of the 2D projection.

#### 2.6.2 Classification-error and neighborhood analysis

The Post-GCTR t-SNE coordinates were annotated with TN, TP, FP, and FN outcomes. The local class purity was computed from the 15 nearest neighbors of each test sample in the Post-GCTR feature space. A neighbourhood was determined to be mixed if there was at least one neighbour with the opposite reference label to that of the test sample. Representative error waveforms were selected based on three pre-specified categories: the probability being closest to the threshold (the so-called boundary error), the highest-confidence errors, and those that were located in a mixed neighbourhood of classes. Sample identifiers were used only to demonstrate that they could be reproduced, and were therefore not included in the labels on the main figure.

#### 2.6.3 Integrated Gradients analysis

The final checkpoints were analyzed using Integrated Gradients (IG) (Sundararajan et al., 2017). In the patient-fixed test set, 50 segments from the normal category and an equal number of segments from the PVC category were selected using the seed 42 so as to ensure representation of both categories. The same 100 sample IDs were used for both methods MS-BLSTM and GCTRNet. A zero-valued ECG segment was used as the baseline, and the same output-logit target was used for both models, with 50 integration steps applied.

A multi-window attribution ratio compiled by use of the ratio of the attribution score of the target beat was computed. For a set time period, the ratio is considered:


$$R_{\text{window}}=\frac{\sum_{t\in \Omega_{\text{window}}}A\left(t\right)}{\sum_{t=1}^{T}A\left(t\right)},$$

where A(t) represents the absolute value of the IG attribution at time t. Therefore, three time windows around the target beat were determined: a narrow window within 0.85-1.15 s, a medium window with 0.75-1.25 s time interval and a wide one with 0.70-1.30 s time period. With respect to the findings obtained, Integrated Gradients were used not as the means of definitive clinical causality but as critical evidence of input level attribution.

### 2.5 External validation

The frozen seed-2024 checkpoints for MS-BLSTM, MS-BLSTM-SE-like, MS-BLSTM-Temporal Attention, ResNet-1D, GCTRNet w/o Gate, and GCTRNet were used to estimate performance on the external dataset. For each model, the checkpoint and operating threshold were selected using only the MITDB validation set and were then held fixed during external evaluation. The validation-selected threshold for GCTRNet was 0.31.

To be able to account for multiple recordings from each patient, 95% confidence intervals for the external performance metrics were obtained from 1,000 bootstrap replicates generated by resampling patients with replacement.

## 3 Results

### 3.1 Controlled comparison with baseline models

The patient-independent MITDB test performance of FCN1D, ResNet1D, MS-BLSTM, MS-BLSTM-SE-like, MS-BLSTM-Temporal Attention, and GCTRNet can be found in **Table 2**; values are reported as means ± standard deviations across seeds 42, 2024, and 3407. The same data split, preprocessing procedure, training conditions, checkpoint selection, and threshold selection were used for all models.

**Table 2:** Internal performance on the patient-independent MITDB test set across three random seeds.

Model	ACC (%)	SEN (%)	SPE (%)	F1-score (%)	AUPRC (%)	MCC	Parameters
FCN1D	92.41 ± 0.33	80.46 ± 5.37	93.52 ± 0.85	64.35 ± 0.68	61.19 ± 3.95	0.6190 ± 0.0127	264,962
ResNet1D	99.85 ± 0.09	98.62 ± 1.10	99.97 ± 0.02	99.13 ± 0.56	99.80 ± 0.25	0.9905 ± 0.0060	3,844,930
MS-BLSTM	98.97 ± 0.46	88.97 ± 4.87	99.90 ± 0.09	93.58 ± 3.03	98.09 ± 1.81	0.9319 ± 0.0313	204,569
MS-BLSTM-SE-like	98.57 ± 0.26	83.76 ± 3.29	99.95 ± 0.05	90.89 ± 1.84	94.23 ± 0.87	0.9053 ± 0.0178	238,681
MS-BLSTM-Temporal Attention	99.10 ± 0.29	94.59 ± 3.34	99.52 ± 0.53	94.73 ± 1.57	99.18 ± 0.12	0.9430 ± 0.0172	271,130
GCTRNet	99.49 ± 0.25	94.44 ± 3.05	99.96 ± 0.05	96.89 ± 1.58	99.27 ± 0.85	0.9666 ± 0.0166	402,714

Performance values are means ± sample standard deviations over seeds 42, 2024, and 3407. For each run, the checkpoint and classification threshold were selected using only the validation set. The test set contained 12,167 normal beats and 1,133 PVCs. The parameter count is invariant across random seeds.

Based on the mean results, ResNet1D had the best mean internal test performance; this included ResNet1D achieving an accuracy of 99.85 ± 0.09%, sensitivity of 98.62 ± 1.10%, F1-score of 99.13 ± 0.56%, and AUPRC of 99.80 ± 0.25%, whereas GCTRNet had an accuracy of 99.49 ± 0.25%, sensitivity of 94.44 ± 3.05%, specificity of 99.96 ± 0.05%, F1-score of 96.89 ± 1.58%, and AUPRC of 99.27 ± 0.85%. Thus, while GCTRNet did not perform better than ResNet1D for internal testing, it performed better than FCN1D and all three BLSTM-based comparison models in terms of mean F1-score.

The number of parameters used in the different models provides context in terms of the differences in model size. GCTRNet had 402,714 parameters, whereas ResNet1D used 3,844,930 parameters, meaning that ResNet1D used approximately 9.55 times as many parameters as GCTRNet. Thus, GCTRNet managed to outperform MS-BLSTM, MS-BLSTM-SE-like, and MS-BLSTM-Temporal Attention with respect to the mean F1-score by 3.31, 6.00, and 2.16 percentage points, respectively, while still using substantially fewer parameters than ResNet1D.

### 3.2 Sensitivity analysis of the BLSTM hidden-state dimension

While D = 128 resulted in a decline in both sensitivity of 67.52% and F1-score of 79.52%, this poor performance indicates insufficient temporal representation capacity. Increasing D to 512 did not increase performance; the sensitivity at D = 512 was 88.53% and F1-score 93.83%, while D = 256 achieved 96.82% sensitivity and 97.90% F1-score. Thus, the intermediate dimension of D = 256 was retained. This experiment provides an internal sensitivity analysis for D. The detailed results are presented in **Table 3**. The external dataset was not used to determine which of the three D value options to select, nor does it make any claims regarding cross-database superiority.

**Table 3:** Sensitivity analysis of the BLSTM hidden-state dimension using seed 2024.

D	Epoch	Val. F1	Threshold	ACC (%)	SEN (%)	SPE (%)	Precision (%)	F1-score (%)	AUPRC (%)	TN/FP/FN/TP
128	20	0.8984	0.16	97.04	67.52	99.79	96.71	79.52	85.30	12,141/26/368/765
256	18	0.9814	0.31	99.65	96.82	99.91	99.01	97.90	99.75	12,156/11/36/1,097
512	12	0.9634	0.12	99.01	88.53	99.98	99.80	93.83	96.23	12,165/2/130/1,003

The data partition, preprocessing, training protocol, and random seed were unchanged across the three settings. The checkpoint and threshold for each dimension were selected using only the validation set. D = 256 provided the best balance of sensitivity, F1-score, and AUPRC.

### 3.3 Ablation study of GCTR

The four GCTR Configurations are recorded in **Table 4** across three different seeds. The LN-only implementation confirmed that the implementation did not contain a global context, adaptive gate, or residual scaling, thus removing any semantic ambiguity present in the no-global case.

**Table 4:** Internal ablation study of GCTR across three random seeds.

Variant	Global context	Adaptive gate	Residual scaling	ACC (%)	SEN (%)	SPE (%)
Full GCTRNet	Yes	Yes	Learnable	99.49 ± 0.25	94.44 ± 3.05	99.96 ± 0.05
Without contextual refinement (LN only)	No	No	N/A	99.18 ± 0.39	92.32 ± 4.65	99.82 ± 0.21
Without adaptive gate	Yes	No	Learnable	99.52 ± 0.08	95.23 ± 0.58	99.92 ± 0.03
Without learnable	Yes	Yes	Fixed at 1	98.94 ± 0.04	89.00 ± 1.41	99.87 ± 0.09

Values are means ± sample standard deviations over seeds 42, 2024, and 3407. LN only denotes post-BLSTM layer normalization without global-context refinement, adaptive gating, or residual scaling.

The full model provided a higher mean accuracy and sensitivity compared to the strict LN-only model: accuracy, 99.49% vs. 99.18%; sensitivity, 94.44% vs. 92.32%. However, this trend was not uniform across the three seeds. Therefore, any isolated contributions provided by global context should be considered seed-dependent.

The elimination of the gate did not reduce internal mean accuracy or sensitivity. The “w/o Gate” variant achieved 99.52 ± 0.08% accuracy and 95.23 ± 0.58% sensitivity, while the original model yielded respective results of 99.49 ± 0.25% and 94.44 ± 3.05%. In contrast, fixing β at 1 reduced mean sensitivity to 89.00 ± 1.41%.

### 3.4 Interpretability analysis

#### 3.4.1 Feature representation analysis

The t-SNE visualization of the pre- and post-GCTR seed-2024 feature representations is shown in **Figure 4**, which also shows the relationship between the pre- and post-GCTR seed-2024 representations via two-dimensional projections. The two projections show only minimal visual differences; therefore, they should not be regarded as concrete evidence of better separation after GCTR. Because of this, an analysis was performed using the original 256-dimensional feature space (**Table 5**).

**Figure 4:**
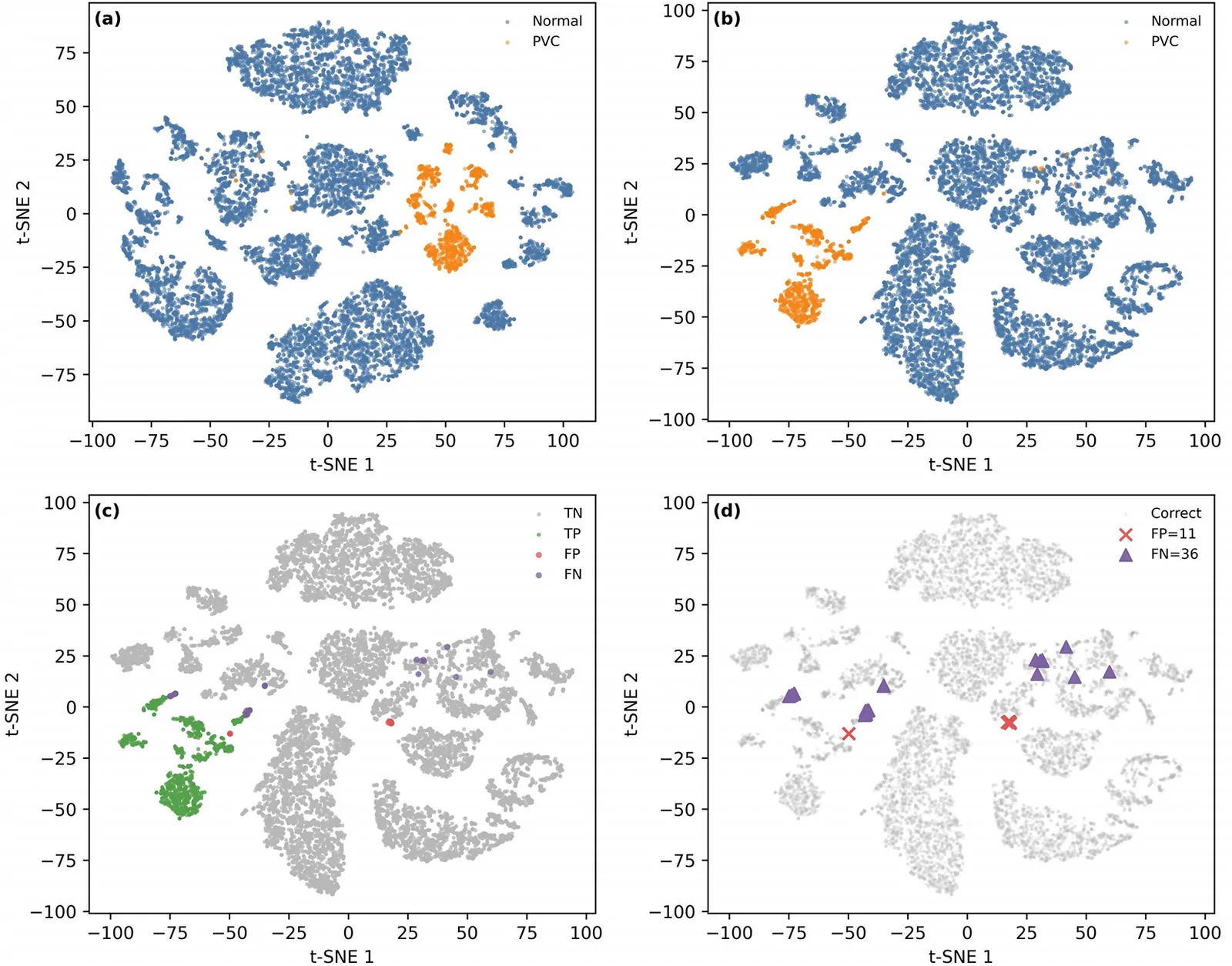
t-SNE visualization of GCTRNet representations and classification outcomes on the patient-fixed MITDB test set. **(a)** Pre-GCTR features colored by reference class. **(b)** Post-GCTR features colored by reference class. **(c)** Post-GCTR features mapped as true negative (TN), true positive (TP), false positive (FP), or false negative (FN). **(d)** The same Post-GCTR embedding with correct predictions shown in light gray and the 11 FPs and 36 FNs from the frozen seed-2024 model highlighted.

**Table 5:** Quantitative comparison of feature compactness and separability before and after GCTR refinement.

Representation	Normalization	Silhouette coefficient	Davies–Bouldin index	Centroid distance	Within-class dispersion	Separation-to-dispersion ratio
Pre-GCTR	Raw	0.430	0.793	2.851	1.131	2.522
Post-GCTR	Raw	0.473	0.695	15.649	5.441	2.876
Pre-GCTR	L2-normalized	0.423	0.744	1.286	0.478	2.688
Post-GCTR	L2-normalized	0.526	0.680	1.410	0.480	2.941
Pre-GCTR	Z-score-normalized	0.321	1.168	21.473	12.541	1.712
Post-GCTR	Z-score-normalized	0.526	0.740	31.167	11.528	2.704

The results of the L2-normalized features show that the silhouette coefficient improved after GCTR (from 0.4229 to 0.5261). The Davies-Bouldin index decreased (from 0.7442 to 0.6800) and the ratio between Centroid Distance and Dispersion increased (from 2.6875 to 2.9412) after GCTR processing. The silhouette coefficient measures how cohesive a particular class is when compared to its closest competing class. The Davies-Bouldin index measures the amount of dispersion within a particular class versus the distance to each of the other classes; it combines these two measurements into one. Centroid Distance indicates the distance between classes; Dispersion indicates the spread of classes; and the ratio of these two values indicates the relative magnitude of each measurement. Therefore, the increase in the silhouette coefficient and the Centroid-Distance-to-Dispersion ratio, along with the decrease in the Davies-Bouldin index, indicates that the classes are better organized in high-dimensional space after GCTR (although visually, on t-SNE, there are minimal differences).

For both the raw and Z-score-normalized representations, the same trends were observed, although the absolute numbers differed because of the way these values were scaled. All metrics described the geometrical configurations of features, but they do not provide evidence of a cause-and-effect relationship or show that all samples would be dramatically separated in the 2D projection. The main evidence for the observed improvements relies solely on the original high-dimensional measurements, rather than any claims about visual patterns observed in the 2D projections.

#### 3.4.2 Classification-error and neighborhood analysis

The frozen seed-2024 GCTRNet displayed a total of 11 false positives and 36 false negatives when evaluated at a validation-derived threshold of 0.31. In the Post-GCTR feature space, it was determined that the mean class-purity values were 0.9999 for true negatives, 0.9985 for true positives, 0.8545 for false positives, and finally 0.6185 for false negatives. Among the 36 false negatives, 11 were located in mixed-class neighborhoods, while only one of the 11 false positives appeared in such neighborhoods; this means that false negatives tended to be linked with a more heterogeneous environment than false positives. Some errors were concentrated in particular recordings, as for instance MITDB recording 207 produced 26 false negatives, while MITDB recording 219 gave rise to 11 false positives and 6 false negatives. Representative classification-error waveforms corresponding to these error patterns are shown in **Figure 5**.

**Figure 5:**
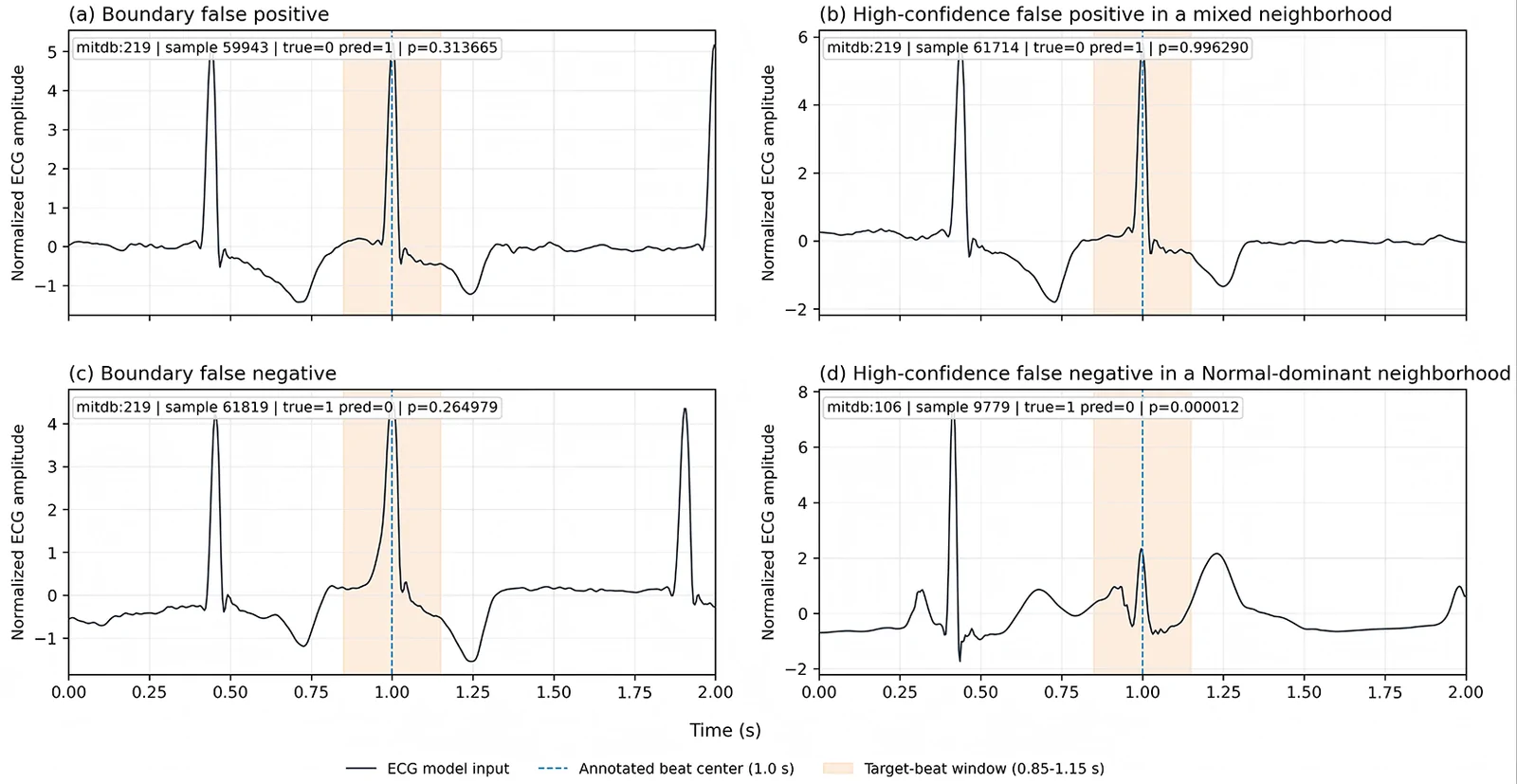
Representative GCTRNet classification-error waveforms on the patient-fixed MITDB test set. **(a)** Boundary false positive. **(b)** High-confidence false positive in a mixed neighborhood. **(c)** Boundary false negative. **(d)** High-confidence false negative in a normal-dominant neighborhood. The shaded region indicates the predefined target-beat window (0.85–1.15 s), and the dashed line marks the annotated beat center at 1.0 s.

#### 3.4.3 Integrated Gradients analysis

**Figure 6** exhibits the attribution patterns of MS-BLSTM and GCTRNet. Strong attribution around the annotated target beat is demonstrated for both models, and GCTRNet shows a broader temporal distribution of attribution. The figure legend details the narrow window used, the center of the annotated beat, the different colors for each model, and indicates that the error bars represent standard deviations.

**Figure 6:**
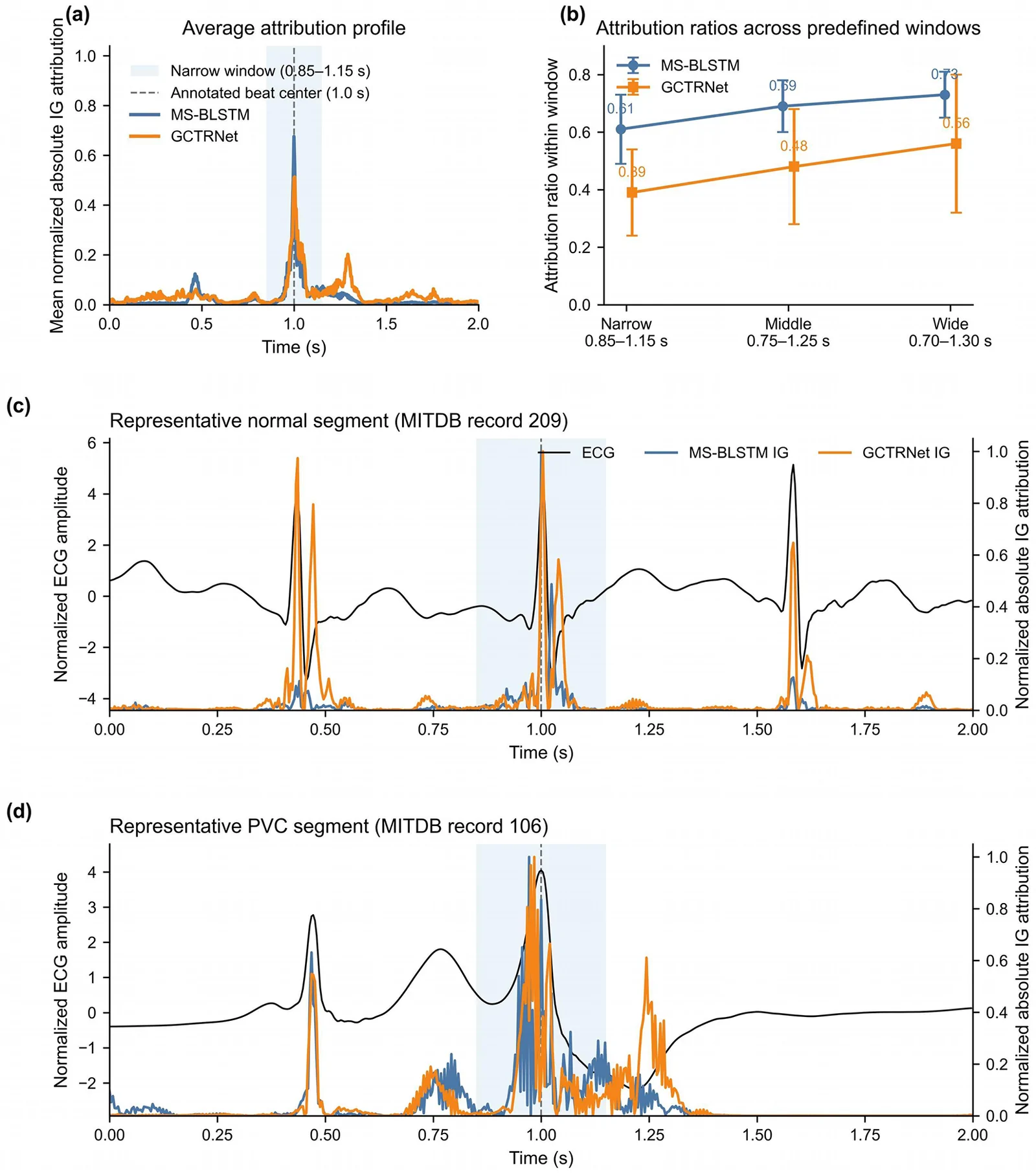
Integrated Gradients analysis of MS-BLSTM and GCTRNet on the patient-fixed MITDB test set. **(a)** Mean normalized absolute IG attribution profiles; the shaded area denotes the narrow window (0.85–1.15 s), and the dashed line marks the annotated beat center at 1.0 s. **(b)** Attribution ratios in the narrow (0.85–1.15 s), middle (0.75–1.25 s), and wide (0.70–1.30 s) windows; error bars show standard deviations. **(c, d)** Representative correctly classified normal and PVC segments, respectively, with the ECG waveform and normalized absolute IG profiles.

The attribution ratios are presented in **Table 6**. The mean attribution ratios across the narrow, medium, and wide windows for MS-BLSTM were 0.61 ± 0.12, 0.69 ± 0.09, and 0.73 ± 0.08, respectively. The corresponding values for GCTRNet were 0.39 ± 0.15, 0.48 ± 0.20, and 0.56 ± 0.24. In essence, GCTRNet yielded lower mean attribution ratios within each analyzed window centered on the target beat, suggesting that it had a broader temporal distribution. The attribution patterns herein should not be interpreted as evidence of physiological causation.

**Table 6:** Integrated Gradients attribution ratios within the predefined temporal windows.

Model	Narrow, 0.85–1.15 s	Medium, 0.75–1.25 s	Wide, 0.70–1.30 s
MS-BLSTM	0.61 ± 0.12	0.69 ± 0.09	0.73 ± 0.08
GCTRNet	0.39 ± 0.15	0.48 ± 0.20	0.56 ± 0.24

Values are means ± standard deviations for the same set of 50 normal and 50 PVC segments. Ratios represent the fraction of total absolute attribution contained within each window.

### 3.5 External validation on a patient-stratified CPSC2021 subset

A locked evaluation set included 210 recordings collected from 105 patients. 209 of these recordings provided valid N/V segments with 392,699 normal segments and 8,313 PVC segments (2.073% PVC prevalence) in total. The six seed-2024 frozen models were evaluated on the same set of recordings with thresholds derived from the MITDB validation dataset. **Table 7** shows the performances of these models.

**Table 7:** Frozen-model external validation on the patient-stratified CPSC2021 subset (seed 2024).

Model	ACC, % (95% CI)	SEN, % (95% CI)	SPE, % (95% CI)	Precision, % (95% CI)	F1-score, % (95% CI)	AUPRC, % (95% CI)
MS-BLSTM	98.44 (96.88–99.42)	54.79 (31.37–87.54)	99.36 (98.77–99.76)	64.43 (39.59–83.67)	59.22 (39.16–78.17)	68.69 (47.33–87.46)
MS-BLSTM-SE-like	96.00 (92.67–97.92)	46.60 (21.33–83.69)	97.05 (94.18–98.64)	25.03 (10.53–45.96)	32.57 (15.50–52.11)	34.78 (13.47–68.49)
MS-BLSTM-Temporal Attention	97.38 (95.39–98.76)	61.52 (35.20–91.46)	98.14 (96.52–99.18)	41.17 (19.80–63.57)	49.33 (28.08–68.40)	54.50 (34.30–76.49)
ResNet1D	73.27 (65.98–81.98)	80.81 (74.34–91.83)	73.11 (65.65–82.07)	5.98 (2.19–13.39)	11.14 (4.27–22.90)	43.62 (21.14–73.21)
GCTRNet w/o Gate	95.92 (92.65–97.83)	71.33 (59.00–91.95)	96.44 (93.32–98.25)	29.80 (13.37–51.44)	42.03 (22.60–59.87)	48.05 (25.78–75.51)
GCTRNet	97.83 (95.89–98.97)	84.75 (77.25–92.16)	98.11 (96.11–99.22)	48.72 (23.43–73.87)	61.87 (36.36–79.15)	80.49 (64.56–88.96)

Checkpoints and thresholds were selected exclusively on MITDB and then frozen. Confidence intervals were estimated using 1,000 patient-level bootstrap resamples. For GCTRNet, TN = 385,284, FP = 7,415, FN = 1,268, and TP = 7,045. For ResNet1D, TN = 287,090, FP = 105,609, FN = 1,595, and TP = 6,718.

GCTRNet obtained accuracy equal to 97.83%, sensitivity equal to 84.75%, specificity equal to 98.11%, precision equal to 48.72%, F1-score equal to 61.87%, and AUPRC equal to 80.49%. GCTRNet achieved the best sensitivity, F1-score, and AUPRC values among all frozen seed-2024 models, while MS-BLSTM achieved the best precision result (64.43%). ResNet1D showed good internal results but produced 105,609 false positives in the external test, thus achieving precision equal to only 5.98% and F1-score equal to 11.14%. Therefore, the difference in model rankings between internal and external evaluation indicates that achieving good performance on MITDB does not guarantee the successful transfer of models to CPSC2021. Under the same framework of frozen seed-2024 external evaluation, full GCTRNet achieved higher sensitivity, F1-score, and AUPRC values than the w/o Gate variant (84.75% against 71.33%, 61.87% against 42.03%, and 80.49% against 48.05%, respectively). The large patient-level bootstrap intervals of sensitivity, precision, and F1-score indicate considerable variation in PVC detection performance across patients.

Overall, these results indicate partial cross-database generalization under the frozen evaluation protocol.

## 4 Discussion

Representative PVC detection methods evaluated on MITDB are summarized in **Table 8**. These studies cover both conventional feature-based approaches and recent deep-learning methods under different experimental designs. Because the underlying datasets and protocols differ, **Table 8** is retained only as descriptive literature context; **Table 2** provides the controlled same-split comparison used for model-level conclusions.

**Table 8:** Contextual summary of representative PVC detection methods evaluated on MITDB.

Study	Year	Method	Experimental design	ACC (%)	SEN (%)	SPE (%)
Xie et al (Xie et al., 2019)	2019	Features + random forest	Inter-patient	96.38	97.88	97.56
Yu et al (Yu et al., 2021)	2021	Deep metric learning + KNN	Intra-patient	99.70	97.45	99.87
Mazidi et al (Mazidi et al., 2022)	2022	TQWT + statistical features + KNN	Intra-patient	97.81	98.23	–
De Marco et al (De Marco et al., 2022)	2022	ML/DL classifiers	Intra-patient	99.90	–	–
Sarshar and Mirzaei (Sarshar and Mirzaei, 2022)	2022	Morphological/statistical features + CNN	Intra-patient	–	94.8	–
Kraft et al (Kraft et al., 2023)	2023	1D U-Net	Inter-patient	–	96.60	92.60
Wang et al (Wang et al., 2024)	2024	PVCNet	Inter-patient	98.50	89.91	–
Ma et al (Ma et al., 2026)	2026	DPFNet	Inter-patient	98.90	97.98	98.97
**Proposed method**	**–**	**GCTRNet**	**Inter-patient**	**99.65**	**96.82**	**99.91**

Because the listed studies used different datasets, patient partitions, preprocessing procedures, class definitions, and evaluation protocols, their reported metrics are provided only for literature context and should not be interpreted as a direct performance comparison with GCTRNet.

Bold values indicate the results of the proposed GCTRNet method.

Under the patient-independent MITDB protocol, the seed-2024 GCTRNet achieved 99.65% accuracy, 96.82% sensitivity, and 99.91% specificity. Across the three seeds, GCTRNet achieved higher mean accuracy, F1-score, and MCC values in comparison with SE-like and temporal-attention counterparts. In contrast to other works, in the current paper GCTRNet does not apply a new temporal encoder or other time-frequency representations but simply refines the hidden states of the BLSTM, still using the original recurrent model. Thus, the effectiveness of the proposed model is supported by the matched model comparisons, component ablation studies, feature-space analysis, and input-attribution evaluations.

However, it is necessary to state that results obtained by comparison of models bear some limitations as many factors such as data partitioning, segmentation strategy, class composition, and evaluation protocols might influence the results reported by different authors. Hence, the primary evidence for relative performance is obtained from the controlled same-split multi-model comparison displayed in **Table 2** as well as the frozen same-sample external comparison given in **Table 7**, as opposed to the ranking of metrics across different studies.

### 4.1 Controlled internal and external comparisons

Based on the results obtained from MITDB, ResNet1D achieved the highest mean accuracy and F1-score among the investigated methods. However, GCTRNet exhibited a different performance trade-off: in terms of mean F1-score, GCTRNet outperformed FCN1D and all three BLSTM-based comparison models. Compared with 3,844,930 for ResNet1D, GCTRNet used only 402,714 parameters. Thus, for internal testing, GCTRNet can be regarded as a competitive, parameter-efficient classifier, rather than the top-performing classifier.

External results on CPSC2021 changed this ranking. ResNet1D exhibits poor transferability, as its high sensitivity is accompanied by a large number of false positives, whereas GCTRNet maintains higher ACC, F1-score, and AUPRC. This performance reversal highlights the importance of frozen, same-sample external comparisons.

Additionally, as demonstrated by hidden-dimension analysis (**Table 3**), larger representation dimensions do not guarantee improved model performance. Specifically, for internal testing, the proposed GCTRNet with D = 256 achieved the highest accuracy, sensitivity, F1-score, and AUPRC among the three tested dimensions.

### 4.2 Scientific insight: why GCTR is suitable for ECG

In contrast to a multi-lead clinical ECG, which contains spatial information across leads, the present research utilizes a selected lead, and hence analyses a temporal waveform derived from only one body surface signal. Evidence of PVC may include QRS duration, amplitude, slope, and overall morphology (Xie et al., 2019; Wang, 2021; Mazidi et al., 2022; Sarshar and Mirzaei, 2022); nevertheless, it may also cover features ranging from the transition into the target beat to the post-QRS waveform and short-term rhythm context (Wang, 2021; Wang et al., 2024). Consequently, the model needs to consider both local morphology and temporal context within the analyzed segment.

The convolutional encoder is good at extracting local forms of respective waveforms, while BLSTM primarily targets various temporal dependencies within the entire segment of ECG. Even after the recurrent encoding, the information contained in the hidden states is still represented across different temporal positions. Temporal pooling techniques usually compress these representations into one vector and may therefore omit information concerning the way contextual features influence individual temporal states.

GCTR is designed to enhance the hidden state sequence by adding contextual information from the entire segment before the classification process. Instead of using an additional temporal encoder, GCTR first summarizes the general information provided by the ECG segment and only then injects this summary information into the temporal sequence.

The presented solution is appropriate for short-window ECG analysis due to the fact that it deals with a limited number of heart cycles in the short segment. Thus, information from the segment as a whole can be used as context for refining individual temporal representations.

### 4.3 Why does GCTR work?

The ablation results further suggest a performance benefit from the GCTR refinement branch. The full GCTRNet achieves higher average accuracy, sensitivity, and specificity than the LN-only model, indicating that the complete GCTR refinement branch contributes to the refinement of the BLSTM representation. The w/o Gate model showed similar average internal performance to the full model, suggesting that the contextual refinement mechanism remains effective even without the adaptive gating component. However, under the frozen seed-2024 evaluation setup, the complete GCTR architecture exhibits higher sensitivity, F1-score, and AUPRC than the w/o Gate variant. This external advantage is in line with the goal of adaptive gating, as it regulates the amount of contextual information at each temporal position.

The contribution of learnable residual scaling was also evident. When β was set equal to 1, the sensitivity decreased the most markedly compared to all other variants tested. This is due to the fact that β controls the strength of the contextual residual update; learning this parameter allows the model to adjust the balance between the initial BLSTM representation and the added contextual representation.

### 4.4 Why interpretability matters

The interpretability analyses shed light on how GCTR changes the representation and ECG information utilization. Although subtle visual differences were shown by the t-SNE plots, the high-dimensional metrics uniformly reflected improved class separability after GCTR. Further neighbourhood analysis demonstrated that false negatives had the lowest local class purity and were more likely to fall within mixed-class regions. As shown by IG analysis, GCTRNet spreads attribution across a wider temporal range than MS-BLSTM, consistent with using segment-level context before pooling. These observations are consistent with the intended representational function of GCTR, but they remain descriptive and fail to establish physiological causality.

### 4.5 External generalization

Even though the dataset CPSC2021 has a lower proportion of PVCs compared to the data set of MITDB (2.073% as opposed to 8.48%), the frozen GCTRNet still managed to obtain a sensitivity of 84.75% and an AUPRC of 80.49% with no retraining on external data and no threshold adjustment. These results indicate that the model is still capable of differentiating PVCs even in a dataset consisting predominantly of normal beats. The precision of 48.72% may be partly related to the application of the threshold selected on MITDB to a dataset consisting predominantly of normal beats; 7,415 false positives occurred despite the low false-positive rate. Therefore, F1-score, AUPRC, and the confusion matrix should be considered in combination with accuracy and specificity. Thus, the obtained results suggest partial generalization to CPSC2021 under the frozen evaluation protocol. The difference from the internal results from the MITDB dataset may be partly accounted for by the difference in hardware that has been used for acquisition, frequency of sampling, characteristics of the lead, noise, annotation methods, and patient population (Wang et al., 2024; Hamami et al., 2026).

### 4.6 Limitations

There are some limitations that should be taken into account. To begin with, external analysis relied on a predetermined subset of CPSC2021, which included at most two recordings per patient rather than all recordings available. Moreover, demographic analysis could not be performed since the publicly available CPSC2021 metadata do not provide information on patient age or sex. Furthermore, weighted cross-entropy was not used for model training. Additionally, the model utilizes only one lead, which prevents any multi-lead spatial analysis from being performed. Finally, the feature and error-waveform analyses are descriptive, and the method requires prior knowledge of beat locations.

### 4.7 Future work

Future research efforts should particularly involve the evaluation of GCTRNet on other complete external cohorts apart from those already evaluated, as well as the exploration of the model’s training and domain-adaptation capabilities through multiple informative data sources. It would also be helpful to assess calibration independently of external test labels. The inclusion of richer demographic information and data on the devices used would allow subgroup and acquisition-specific analyses. Also, it would be a major step towards putting the solution into practice to broaden the model use from segment-level classification to providing joint beat localization and classification and assessing multi-lead channels for deployment with wearable devices and Holter recording systems.

## 5 Conclusion

GCTRNet incorporates a gated residual update that uses segment-level context to refine individual BLSTM temporal states, facilitating the representation of different parts of a sequence. GCTRNet achieved a higher mean F1-score compared with FCN1D and three other BLSTM-based models across the three random seeds when tested on the patient-independent MITDB dataset. Even though ResNet1D gave the best mean internal accuracy and F1-score, its parameter count was approximately 9.55 times that of GCTRNet. When the frozen GCTRNet model was applied directly to the CPSC2021 dataset using its MITDB-derived threshold, it achieved the best F1-score and AUPRC among the models tested. Despite the performance drop observed from internal to external validation, the results generally suggest that GCTR can refine BLSTM representations for short-window PVC detection in a parsimonious manner and pave the way for further studies using broader external datasets.

## Data Availability

Publicly available datasets were analyzed in this study. This data can be found here: MIT-BIH Arrhythmia Database, PhysioNet, version 1.0.0: https://physionet.org/content/mitdb/1.0.0/. DOI: 10.13026/C2F305. China Physiological Signal Challenge 2021 (CPSC2021), PhysioNet, version 1.0.0: https://physionet.org/content/cpsc2021/1.0.0/.
